# Postoperative opioid-free analgesia with acetaminophen and its impact on pain and postoperative nausea and vomiting following arthroscopic rotator cuff repair: a single-center retrospective study

**DOI:** 10.1186/s40780-025-00530-6

**Published:** 2025-12-22

**Authors:** Misa Okizuka, Ryo Inose, Shiho Maruoka, Mikiko Takeuchi, Subaru Hirotsu, Miki Nakamura, Yuichi Muraki

**Affiliations:** 1 Pharmaceutical Department, Rakuwakai Marutamachi Hospital, 9-7 Matsushita-cho Jurakumawari, Nakagyo-ku, Kyoto, 604-8401 Japan; 2 Laboratory of Clinical Pharmacoepidemiology, Kyoto Pharmaceutical University, 5 Misasagi-Nakauchi-cho, Yamashina-ku, Kyoto, 607-8414 Japan; 3 Department of Anesthesiology, Rakuwakai Marutamachi Hospital, 9-7 Matsushita-cho, Jurakumawari, Nakagyo-ku, Kyoto, 604-8401 Japan; 4 Surgical Center, Rakuwakai Marutamachi Hospital, 9-7 Matsushita-cho, Jurakumawari, Nakagyo-ku, Kyoto, 604-8401 Japan; 5 Rehabilitation Department, Rakuwakai Marutamachi Hospital, 9-7 Matsushita-cho, Kyoto, 604-8401 Japan

**Keywords:** Multimodal analgesia, Postoperative nausea and vomiting, Acetaminophen, Numerical rating scale, Opioid-free analgesia

## Abstract

**Background:**

Opioids are commonly used for postoperative analgesia after arthroscopic rotator cuff repair; however, they pose an increased risk of postoperative nausea and vomiting. Therefore, multimodal analgesia using non-opioid analgesics is recommended. However, the efficacy of postoperative opioid-free analgesia, including acetaminophen, has not been fully elucidated. Therefore, this study aimed to assess the effect of postoperative opioid-free, acetaminophen-containing analgesia on postoperative outcomes in patients undergoing arthroscopic rotator cuff repair.

**Methods:**

Patients who underwent arthroscopic rotator cuff repair at Rakuwakai Marutamachi Hospital between June 2022 and May 2025 were included. The patients were divided into postoperative opioid and opioid-free groups. Propensity score matching was used to balance baseline characteristics. The postoperative outcomes, including pain scores, incidence of nausea and vomiting, use of additional analgesics, and length of postoperative hospital stay, were compared between the groups.

**Results:**

After propensity score matching, 78 patients were assigned to each group. Postoperative pain scores did not significantly differ between the groups. However, the postoperative opioid-free group had a significantly lower incidence of nausea and vomiting (5.1% vs. 20.5%, *p* < 0.05) and use of additional analgesics (5.1% vs. 20.5%, *p* < 0.05). The length of postoperative hospital stay did not differ significantly between the groups (13 days vs. 11.5 days, *p* = 0.268).

**Conclusions:**

Postoperative opioid-free analgesia, including acetaminophen, may provide pain control comparable to conventional opioid-based strategies, reduce the incidence of postoperative nausea and vomiting, and represent a safe and effective approach for postoperative analgesia after arthroscopic rotator cuff repair.

**Supplementary Information:**

The online version contains supplementary material available at 10.1186/s40780-025-00530-6.

## Background

Arthroscopic rotator cuff repair is now widely performed as a standard treatment for rotator cuff tears [1], and recent epidemiological studies have demonstrated a continuous increase in the number of procedures performed over the past decade, owing to its minimally invasive nature and high functional outcomes [2]. However, severe postoperative pain commonly occurs, and inadequate analgesia may delay the initiation of rehabilitation, leading to long-term functional impairment [3]. According to previous reports, early postoperative pain is a risk factor for shoulder stiffness after arthroscopic rotator cuff repair [4]. If shoulder stiffness is not appropriately treated, it can cause significant functional limitations, including restricted range of motion and chronic pain [5]. Hence, proper analgesia following arthroscopic rotator cuff repair is critical to achieve a favorable postoperative course and functional recovery.

Opioids are commonly used for postoperative analgesia following arthroscopic rotator cuff repair [6]. Although effective for analgesia, opioids are frequently associated with postoperative nausea and vomiting (PONV) [7], which is an unpleasant and distressing symptom that significantly affects recovery [8]. Therefore, multimodal analgesia (MMA), aimed at providing effective pain relief while minimizing opioid use, has gained attention and is recommended by clinical guidelines [9].

By combining multiple non-opioid analgesics with different mechanisms of action, MMA enhances analgesic efficacy while reducing side effects compared with opioid monotherapy. It is widely used in the perioperative management of various surgeries [10]. Introduction of MMA has been reported to significantly reduce opioid consumption [11–13]. However, the selection of drugs and routes of administration within MMA is considerably diverse, and standardized protocols supported by sufficient evidence have yet to be established.

Among the non-opioid agents commonly used in MMA, those with sufficient evidence of efficacy include gabapentinoids, acetaminophen, ketamine, nonsteroidal anti-inflammatory drugs, and local anesthetics [14]. In particular, acetaminophen is noteworthy for its high safety profile, central analgesic action, and ability to reduce postoperative opioid use [15, 16]. However, most of these findings were derived from opioid-incorporating MMA, and the efficacy and safety of postoperative opioid-free MMA, including that containing acetaminophen, remain unclear.

This study aimed to clarify the efficacy and safety of postoperative opioid-free, acetaminophen-containing MMA in patients who underwent arthroscopic rotator cuff repair.

## Methods

### Study design and participants

This retrospective cohort study was conducted at Rakuwakai Marutamachi Hospital in Kyoto Prefecture and included patients who underwent arthroscopic rotator cuff repair between June 2022 and May 2025. Based on postoperative opioid use, the patients were assigned to either the opioid group (patients who received opioids after surgery between June 2022 and May 2023) or the postoperative opioid-free group (patients who did not receive opioids after surgery between June 2023 and May 2025). Patients who did not receive a prespecified analgesic regimen were excluded. Tramadol, which is not classified as a narcotic under the Narcotics and Psychotropics Control Law, was administered to both groups and was considered separately from the other narcotics.

### Predetermined analgesic schedule

**Fig. 1:**
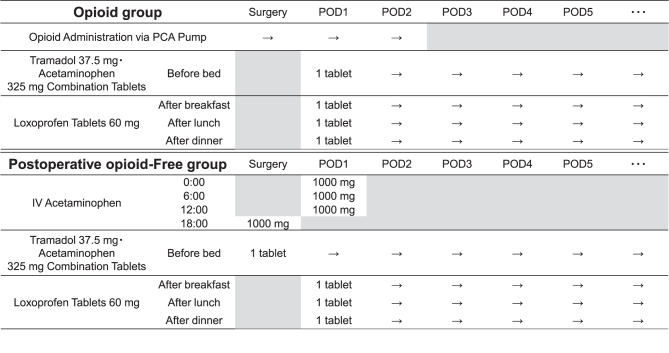
Postoperative analgesic administration schedule for opioid and postoperative opioid-free groups. Abbreviations: pod, postoperative day; IV, intravenous; PCA, patient-controlled analgesia; IV, acetaminophen, 1000 mg every 6 h from 18:00 to 12:00 (500 mg if < 50 kg)

Postoperative analgesics were administered according to the schedule shown in Fig. 1. The opioid group received continuous intravenous fentanyl via a patient-controlled analgesia (PCA) pump (Vessel Fuser, Toray Medical Co., Ltd.) in the operating room, and the PCA concentration and optional droperidol were determined by the anesthesiologist. Loxoprofen (60 mg) was orally administered after each meal starting on postoperative day 1, and tramadol 37.5 mg/acetaminophen 325 mg combination tablet was orally administered before bedtime. The postoperative opioid-free group was administered intravenous acetaminophen (1000 mg) every 6 hours from 18:00 on the day of surgery until 12:00 the next day (500 mg for patients weighing < 50 kg), followed by an oral tramadol/acetaminophen tablet before bedtime on the day of surgery and loxoprofen (60 mg) after each meal from postoperative day 1. The postoperative opioid-free analgesic regimen was determined through consultation with anesthesiologists, nurses, and pharmacists on the acute pain service team in collaboration with the attending orthopedic surgeon. Patient allocation to the opioid-free group was not randomized; rather, it followed an institutional protocol applied to all patients who underwent arthroscopic rotator cuff repair from June 2023 onwards. In both groups, the discontinuation of regular oral analgesics for pain relief was prearranged by an agreement between the attending physician and pharmacist that the pharmacist could discontinue the medications. Discontinuation of oral analgesics was determined by a pharmacist after consultation with the patient. Additional analgesics or antiemetics were administered in the postoperative ward according to patient symptoms and Numeric Rating Scale (NRS) scores after discussion among orthopedic surgeons, nurses, and pharmacists.

### Outcomes and data collection

A retrospective chart review was performed to collect the patients’ background information. Baseline variables included sex, age, body mass index (BMI), American Society of Anesthesiologists physical status (ASA-PS) classification, tear size (small, moderate, large, massive, or other), smoking status (smoker or non-smoker), and history of PONV prior to surgery. Laboratory data included creatinine clearance (CCr), aspartate aminotransferase (AST), alanine aminotransferase (ALT), and γ-glutamyl transpeptidase (γ-GTP). Surgical and anesthetic data included the operative time, intraoperative opioid use (fentanyl and remifentanil), volatile anesthetic use (sevoflurane and desflurane), local anesthetic use in the operating room (ropivacaine), intraoperative analgesic use (flurbiprofen and acetaminophen), and intraoperative antiemetic use (dexamethasone, droperidol, metoclopramide, ondansetron, and granisetron).

The primary endpoint was the NRS scores on postoperative days 1 to 5. The secondary endpoints were the incidence of PONV, postoperative antiemetic use, fentanyl dose administered via a PCA pump, PCA discontinuation, reasons for discontinuation (PONV, adequate pain control, or other reasons), fentanyl wastage following discontinuation, additional postoperative analgesic use, discontinuation of or reduction in regular oral analgesics during hospitalization, number of days from surgery (day 0) to the first discontinuation of or reduction in regular oral analgesics, and the length of postoperative hospital stay, with all outcomes assessed in the postoperative ward.

Clinical data, including NRS scores, were collected to the greatest extent possible, even if partially missing. Postoperative NRS scores were recorded by ward nurses during morning vital sign measurements and, unless otherwise noted, represented pain at rest. When multiple scores were recorded on the same postoperative day, the maximum score was used as the representative value for analysis. PONV was assessed based on patient reports and records of antiemetic administration, both of which were documented in the electronic medical records. For PCA discontinuation, cases in which the reason could not be determined to be either PONV or adequate pain control or was clearly attributable to other factors were categorized as “other reasons.”

### Statistical analyses

Statistical analyses were conducted using R software (version 4.5.1; R Foundation for Statistical Computing, Vienna, Austria) with the R Commander package (version 2.9.5). Propensity score matching was used to balance baseline characteristics between the opioid and postoperative opioid-free groups. Propensity scores were estimated using logistic regression, and 1:1 nearest-neighbor matching was performed with a caliper width of 0.2. The qualitative variables included sex, ASA-PS classification, tear size, non-smoking status, history of PONV, intraoperative analgesics (flurbiprofen and acetaminophen) administered in the operating room, and intraoperative antiemetics (dexamethasone, droperidol, ondansetron, and granisetron). Quantitative variables included age, BMI, weight, CCr, AST, ALT, γ-GTP, operative time, intraoperative opioid (fentanyl and remifentanil) consumption, volatile anesthetic (sevoflurane and desflurane) consumption, intraoperative local anesthetic (ropivacaine) consumption. Metoclopramide was excluded from the matching variables because it was rarely administered in either group.

Covariate balance before and after matching was assessed using the standardized mean difference (SMD), with adequate balance indicated by an SMD < 0.10. After matching, the outcomes compared between the groups included the incidence of PONV, postoperative antiemetic use, additional postoperative analgesic use, and discontinuation or reduction in scheduled oral analgesics, and were analyzed using Fisher’s exact test, where appropriate. PCA discontinuation and its reasons, as well as fentanyl dosage and discarded fentanyl, were analyzed only in the opioid group. NRS scores, time to first discontinuation or reduction of oral analgesics, and length of postoperative hospital stay were compared using the Mann–Whitney U test. Sensitivity analysis was performed to assess the robustness of the results. In addition to the propensity score-matched cohort in the main analysis, the same statistical tests were applied to the unadjusted pre-matched data, and the same outcome measures were evaluated. All tests were two-tailed, and statistical significance was set at *p* < 0.05.

## Results

### Comparison of postoperative NRS scores between the opioid and postoperative opioid-free groups

**Fig. 2:**
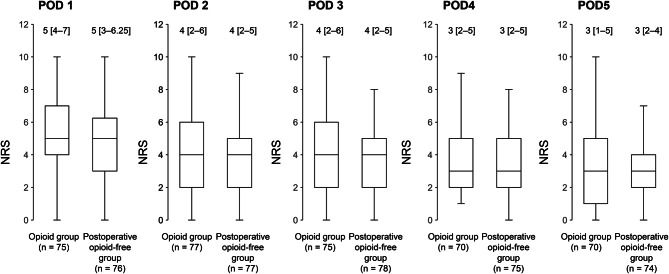
Postoperative NRS scores on days 1–5 after propensity score matching. Data are presented as the median [interquartile range]. Statistical significance was assessed using the Mann–Whitney U test (* *p* < 0.05). POD, postoperative day

In this study, 421 patients were screened, and 57 who did not receive a predetermined analgesic regimen were excluded. Ultimately, 364 patients were included in the final analysis. The patients were divided into an opioid group (*n* = 102) and a postoperative opioid-free group (*n* = 262) before propensity score matching (Table 1). After propensity score matching, patient characteristics were generally balanced between the opioid and postoperative opioid-free groups, with 78 patients included in each group for analysis (Table 1). The median NRS scores from postoperative days 1 to 5 were not significantly different between the two groups at any time point (Fig. 2).

**Table 1:** Comparison of baseline patient characteristics before and after propensity score matching according to analgesic protocol change

	Before propensity score matching			After propensity score matching		
	Opioid group (*n* = 102)	Postoperative opioid-free group (n = 262)	SMD	Opioid group (n = 78)	Postoperative opioid-free group (n = 78)	SMD
Sex (female)^a^	33 (32.4)	115 (43.9)	0.239	28 (35.9)	35 (44.9)	0.184
Age (years)^b^	67.0 [59.3–72.0]	66.0 [57.3–73.0]	0.097	66.0 [58.0–72.0]	67.0 [59.0–74.0]	0.028
BMI(kg/m^2^)^b^	24.0 [21.1–26.0]	24.0 [21.6–26.3]	0.063	23.9 [21.0–26.0]	24.0 [21.6–26.4]	0.021
ASA-PS Classification			0.077			< 0.001
ASA-PS1^a^	36 (35.3)	83 (31.7)		27 (34.6)	27 (34.6)	
ASA-PS2^a^	61 (59.8)	166 (63.4)		48 (61.5)	48 (61.5)	
ASA-PS3^a^	5 (4.9)	13 (5.0)		3 (3.8)	3 (3.8)	
tear size			0.332			0.264
small^a^	11 (10.8)	57 (21.8)		10 (12.8)	12 (15.4)	
moderate^a^	30 (29.4)	65 (24.8)		20 (25.6)	19 (24.4)	
large^a^	22 (21.6)	43 (16.4)		18 (23.1)	14 (17.9)	
massive^a^	20 (19.6)	58 (22.1)		16 (20.5)	12 (15.4)	
other^a^	19 (18.6)	39 (14.9)		21 (26.9)	14 (17.9)	
AST (U/L)^b^	22.0 [19.0–26.0]	23.0 [19.0–27.0]	0.107	22.5 [19.0–27.8]	23.0 [20.3–26.8]	0.001
ALT (U/L)^b^	21.5 [17.3–28.0]	20.5 [16.0–28.8]	0.090	23.0 [17.3–30.0]	21.0 [17.0–28.0]	0.169
γ-GTP (U/L)^b^	29.5 [19.5–46.0]	26.0 [18.0–44.0]	0.065	31.0 [18.3–47.8]	26.0 [18.0–39.8]	0.357
CCr(mL/min)^b^	80.8 [64.2–99.6]	79.5 [65.5–95.2]	0.100	79.9 [64.2–98.5]	82.2 [69.6–96.7]	0.011
Smoking status (non-smoker)^a^	74 (73.3)	218 (83.5)	0.251	58 (74.4)	61 (78.2)	0.091
History of PONV^a^	6 (6.3)	27 (11.2)	0.172	5 (6.4)	5 (6.4)	< 0.001
Operation time (min)^b^	114.0 [94.3–156.0]	102.5 [69.0–145.5]	0.275	112.5 [92.3–148.5]	106.0 [71.3–151.5]	0.121
Intraoperative opioid use						
Remifentanil (μg)^b^	433.0 [304.8–659.3]	331.0 [215.0–530.0]	0.159	401.5 [296.5–617.8]	382.0 [219.5–533.8]	0.106
Fentanyl (μg)^b^	150.0 [100.0–200.0]	150.0 [100.0–200.0]	0.362	150.0 [100.0–200.0]	150.0 [100.0–200.0]	0.007
Volatile anesthetic use						
Sevoflurane (ml)^b^	43.0 [30.9–58.2]	38.0 [25.3–52.4]	0.118	43.6 [31.6–59.3]	38.9 [26.8–53.5]	0.177
Desflurane (ml)^b^	0.0 [0.0–0.0]	0.0 [0.0–0.0]	0.007	0.0 [0.0–0.0]	0.0 [0.0–0.0]	0.095
Local anesthetic use						
Ropivacaine (mg)^b^	82.5 [75.0–112.5]	90.0 [75.0–112.5]	0.304	90.0 [75.0–112.5]	90.0 [75.0–112.5]	0.129
Intraoperative analgesic use						
Flurbiprofen^b^	78 (76.5)	175 (66.8)	0.216	57 (73.1)	56 (71.8)	0.029
Acetaminophen^b^	9 (8.8)	40 (15.3)	0.199	9 (11.5)	7 (11.0)	0.085
Intraoperative antiemetic use						
Dexamethasone^b^	87 (85.3)	180 (68.7)	0.402	66 (84.6)	63 (80.8)	0.102
Droperidol^b^	82 (80.4)	211 (80.5)	0.004	63 (80.8)	63 (80.8)	< 0.001
Metoclopramide^b^	0 (0.0)	0 (0.0)		0 (0.0)	0 (0.0)	
Ondansetron^b^	7 (6.9)	21 (8.0)	0.044	5 (6.4)	5 (6.4)	< 0.001
Granisetron^b^	0 (0.0)	21 (8.0)	0.417	0 (0.0)	0 (0.0)	0.000

SMD, standardized mean difference; BMI, Body Mass Index; ASA-PS, American Society of Anesthesiologists Physical status; AST, Serum aspartate aminotransferase; ALT, alanine aminotransferase; γ-GTP, γ-glutamyl transpeptidase; CCr, creatinine clearance; PONV, Postoperative nausea and vomiting a Data are expressed as n (%). b Data are expressed as median [interquartile range]

### Incidence of PONV and Opioid administration via PCA pump

PONV occurred in 16 patients (20.5%) in the opioid group and 4 patients (5.1%) in the postoperative opioid-free group, indicating significantly lower occurrence in the latter group (*p* < 0.05) (Table 2). No significant differences in antiemetic use were observed in the postoperative ward. Among the patients in the opioid group, 33 (42.3%) completed PCA administration, whereas 45 (57.7%) discontinued PCA prematurely. The reasons for discontinuation were PONV in 14 (31.1%) and adequate pain control in 20 patients (44.4%). The median fentanyl dose administered via PCA pump was 900 µg, and the median amount discarded due to premature discontinuation was 320 µg.

**Table 2:** Incidence of PONV and Opioid administration via PCA pump

	Opioid group (n = 78)	Postoperative opioid-free group (n = 78)	p-value
Incidence of PONV			
Incidence of PONV	16 (20.5)	4 (5.1)	< 0.05^a)^
Postoperative antiemetic use	10 (12.8)	5 (6.4)	0.227^a)^
Metoclopramide	10 (100.0)	3 (38.5)	
Domperidone	0 (0.0)	1 (12.8)	
Ondansetron	0 (0.0)	0 (0.0)	
Granisetron	0 (0.0)	1 (12.8)	
Opioid Administration via PCA Pump			
Completion of administration	33 (42.3)	N/A	
Discontinuation during treatment	45 (57.7)	N/A	
Discontinuation due to PONV	14 (31.1)	N/A	
Discontinuation due to adequate pain control	20 (44.4)	N/A	
Discontinuation for reasons other than the above	11 (24.4)	N/A	
Fentanyl dose administered via PCA pump (μg) Fentanyl waste volume (μg)	900 [800–1000]	N/A	
	320 [95–550]	N/A	

PCA, patient-controlled analgesia; PONV, postoperative nausea and vomiting. Data are expressed as n (%); N/A, not applicable; ^a^ Fisher’s exact test

### Postoperative analgesic use and length of hospital stay

In the postoperative ward, additional analgesics were administered to 16 (20.5%) and 4 patients (5.1%) in the opioid and postoperative opioid-free groups, respectively, indicating significantly less frequent occurrence in the latter group (*p* < 0.05) (Table 3). Regular oral analgesics were discontinued or reduced during hospitalization in 56 patients (71.8%) in the opioid group and in 46 patients (59.0%) in the postoperative opioid-free group, with no significant difference between the groups. Furthermore, the median time to the first discontinuation or reduction in regular oral analgesics (considering the day of surgery as day 0) and length of postoperative hospital stay were not significantly different between the two groups.

**Table 3:** Postoperative analgesic use and length of postoperative hospital stay

	Opioid group (*n* = 78)	Postoperative opioid-free group (*n* = 78)	p-value
Postoperative Analgesic Use			
Additional analgesic use	16 (20.5)	4 (5.1)	< 0.05^a)^
Discontinuation or reduction of regular oral analgesics prior to discharge	56 (71.8)	46 (59.0)	0.272^a)^
Days to first reduction of regular oral analgesics	6.5 [5.0–8.0]	7.0 [5.5–8.0]	0.247^b)^
Other			
Length of postoperative hospital stay	13.0 [7.0–19.0]	11.5 [6.0–16.0]	0.268^b)^

Values are presented as numbers (percentages). For continuous variables, data are shown as median [interquartile range]. ^a)^ Fisher’s exact test; ^b)^ Mann–Whitney U test

### Sensitivity analysis

When the same statistical tests were applied to the unadjusted data before propensity score matching (*n* = 364), no significant differences were observed in postoperative NRS scores (Supplemental Fig. S1) or length of postoperative hospital stay (15.0 days vs. 14.0 days, *p* = 0.134). However, the incidence of PONV (8.0% vs. 24.5%, *p* < 0.05) and frequency of additional analgesic use (5.6% vs. 21.6%, *p* < 0.05) were significantly lower in the postoperative opioid-free group.

## Discussion

In a real-world clinical setting, our findings suggest that postoperative opioid-free MMA in arthroscopic rotator cuff repair may reduce the incidence of PONV, the need for additional analgesics, and drug wastage, although the pain scores did not differ significantly between the groups.

The postoperative NRS scores of the two groups were not significantly different. In the opioid group, 42.3% of the patients completed PCA, whereas 57.7% discontinued PCA prematurely. The main reasons for discontinuation were PONV (31.1%) and achievement of adequate pain control (44.4%). After the discontinuation of PCA, analgesics were administered intermittently rather than continuously. This may have led to less stable pain control and greater variability in pain intensity among the patients. Such variability, combined with potential opioid-induced hyperalgesia mediated by reduced β-endorphin production and μ-opioid receptor downregulation [17], may have attenuated the apparent differences in mean pain scores between groups, making it more difficult to detect statistically significant differences. Although preclinical studies have suggested mechanisms, such as N-methyl-D-aspartate receptor activation, impaired descending pain inhibition, and neuroinflammatory responses, clinical studies directly examining these mechanisms remain limited [18], and the precise underlying pathways remain unclear.

Furthermore, as shown in Fig. 2, a considerable number of patients in both groups had NRS scores > 4 in the early postoperative period. This does not indicate inadequate pain management, but rather reflects the inherently high level of acute postoperative pain following shoulder surgery. A previous study also reported that many patients experienced significant pain in the early phase after rotator cuff repair [4], and our results are generally consistent with this finding. The inherently high level of acute postoperative pain following shoulder surgery may have masked subtle differences between the groups.

The incidence of PONV and frequency of additional postoperative analgesic use were significantly lower in the postoperative opioid-free group, with identical values of 5.1% and 20.5%, respectively (*p* < 0.05). Postoperative opioid use is a well-established, dose-dependent risk factor for PONV [7], and the higher incidence of PONV observed in the opioid group in this study was an expected finding. In contrast, the frequency of additional analgesic use itself did not increase PONV; rather, early discontinuation of PCA due to PONV likely led to the increased administration of additional analgesics in the opioid group. Moreover, because additional analgesics can temporarily suppress acute pain peaks, their use may have contributed to the relatively lower mean NRS scores in the opioid group, potentially further attenuating between-group differences. These results support the safety and efficacy of non-opioid-centered MMA and are consistent with previous findings demonstrating the safety and efficacy of acetaminophen-based opioid-free MMA after total hip arthroplasty [19]. For sensitivity analysis, we analyzed the unadjusted data before propensity score matching. No significant differences were observed between the groups in the postoperative NRS scores, whereas the incidence of PONV and the frequency of additional analgesic use were significantly lower in the postoperative opioid-free group, concurrent with the primary analysis. These findings confirm the robustness of our results.

Primary and sensitivity analyses of the postoperative length of stay revealed no significant differences between the groups. Discharge following shoulder surgery necessitates independence in activities of daily living, including dressing, eating, personal hygiene, and toileting [20]; therefore, differences in the incidence of PONV or additional analgesic use alone may not have exerted a statistically significant impact on the length of stay. In contrast, the postoperative opioid-free group experienced practical advantages beyond the clinical aspects. Specifically, the group did not generate fentanyl waste, which may have reduced the disposal-associated operational burden, such as the requirements for witnessing and record keeping. The temporal and human costs associated with narcotic disposal have been reported as a significant burden for healthcare professionals [21]. Therefore, the findings of this study are considered significant not only for their clinical utility but also for improving operational efficiency.

This study has several limitations. First, the study was conducted at a single institution with a limited number of cases, and caution is required when generalizing the results. Second, although propensity score matching improved the overall balance of patient characteristics, some factors—such as tear size and γ-GTP—remained imbalanced between groups, indicating that complete adjustment for confounding was not achieved. Therefore, the influence of unadjusted or residual confounding factors cannot be completely excluded. Third, the outcome assessments relied on routine clinical documentation rather than on standardized protocols. For example, NRS scores were recorded at various times, and PONV was assessed based on patient reports and antiemetic administration records. These factors may have led to the incomplete capture of acute pain fluctuations and underestimation of mild nausea. Despite these limitations, our findings suggest that postoperative opioid-free analgesia during shoulder surgery is not inferior to conventional opioid-based analgesia. This approach may reduce the incidence of PONV and the need for supplemental analgesics, potentially leading to a decreased patient burden and enhanced safety. This study is unique because it presents drug waste data, thereby offering a new perspective on the practical aspects of postoperative analgesia. Reports addressing the operational considerations and clinical aspects of postoperative analgesia are limited, and this study contributes to filling this gap. A study that confirms both safety and efficacy while demonstrating operational advantages that align with previous findings holds significant value. Future research, including a quantitative evaluation of the operational burden, is warranted.

## Conclusion

This study demonstrated that postoperative opioid-free analgesia significantly reduced the incidence of PONV and the use of additional analgesics compared with conventional opioid-based analgesia. These findings provide valuable insights that may broaden postoperative analgesia strategies in orthopedic surgery and contribute to reducing the patient burden.

## Electronic supplementary material

Below is the link to the electronic supplementary material.

Supplementary material 1

## Data Availability

The datasets generated and/or analyzed in the current study are available from the corresponding author upon reasonable request.

## Abbreviations

ALT: Alanine Aminotransferase
ASA-PS: American Society of Anesthesiologists Physical Status
AST: Aspartate Aminotransferase
BMI: Body Mass Index
CCr: Creatinine Clearance
MMA: Multimodal Analgesia
NRS: Numeric Rating Scale
PONV: Postoperative Nausea and Vomiting
PCA: Patient-Controlled Analgesia
SMD: Standardized Mean Difference
γ-GTP γ: -Glutamyl Transpeptidase
