# Supplementary material for: Postoperative opioid-free analgesia with acetaminophen and its impact on pain and postoperative nausea and vomiting following arthroscopic rotator cuff repair: a single-center retrospective study
Source: J Pharm Health Care Sci. 2025 Dec 22;11:115. doi: 10.1186/s40780-025-00530-6 (PMC12751185; doi:10.1186/s40780-025-00530-6)
Supplement: Supplementary file 1 — Supplementary material 1 [file 40780_2025_530_MOESM1_ESM.pptx]

## Slide 1
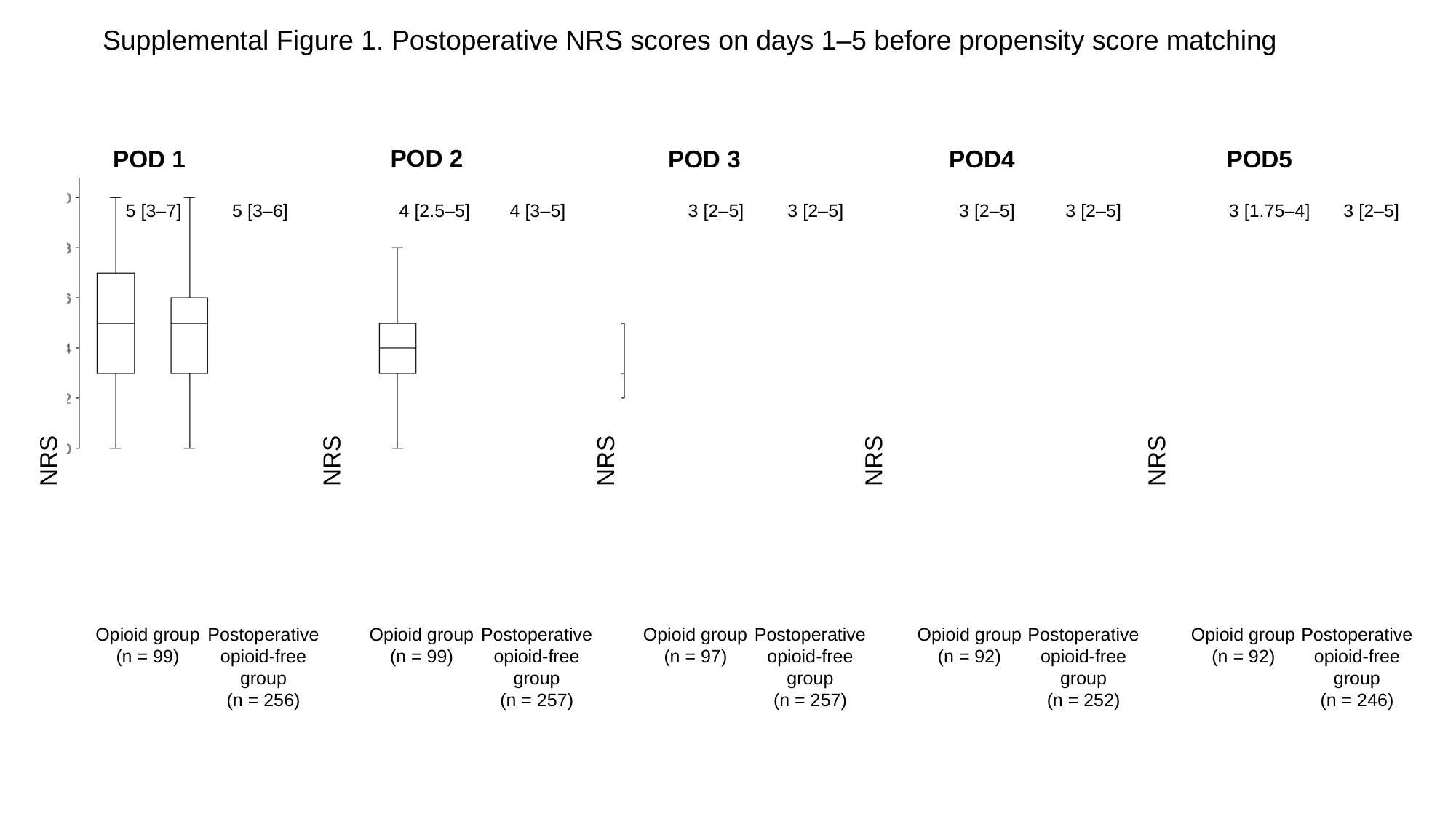

Supplemental Figure 1. Postoperative NRS scores on days 1–5 before propensity score matching
POD 2
POD 1
POD 3
POD4
POD5
5 [3–7]
5 [3–6]
4 [2.5–5]
4 [3–5]
3 [2–5]
3 [2–5]
3 [2–5]
3 [2–5]
3 [1.75–4]
3 [2–5]
NRS
NRS
NRS
NRS
NRS
Opioid group
(n = 99)
Postoperative opioid-free group
(n = 256)
Opioid group
(n = 99)
Postoperative opioid-free group
(n = 257)
Opioid group
(n = 97)
Postoperative opioid-free group
(n = 257)
Opioid group
(n = 92)
Postoperative opioid-free group
(n = 252)
Opioid group
(n = 92)
Postoperative opioid-free group
(n = 246)
